# Multifactorial Landscape Parses to Reveal a Predictive Model for Knee Osteoarthritis

**DOI:** 10.3390/ijerph18115933

**Published:** 2021-05-31

**Authors:** Monica Singh, Srishti Valecha, Rubanpal Khinda, Nitin Kumar, Surinderpal Singh, Pawan K. Juneja, Taranpal Kaur, Mario Di Napoli, Jatinder S. Minhas, Puneetpal Singh, Sarabjit Mastana

**Affiliations:** 1 Division of Molecular Genetics, Department of Human Genetics, Punjabi University, Patiala 147002, India; singhmonica2017@gmail.com (M.S.); srishti_rs19@pbi.ac.in (S.V.); ruban_rs19@pbi.ac.in (R.K.); nitin_rs19@pbi.ac.in (N.K.); 2 Aggarwal Orthopedic Hospital, Ludhiana 141001, India; spsaoh@gmail.com (S.S.); pwkjaoh2016@gmail.com (P.K.J.); 3 Amrit Sagar Hospital, Ferozepur 152001, India; tpashf2012@gmail.com; 4 Neurological Service, Annunziata Hospital, Sulmona, 67039 L’Aquila, Italy; mariodinapoli@katamail.com; 5 Department of Cardiovascular Sciences, University of Leicester, Leicester LE1 7RH, UK; jm591@leicester.ac.uk; 6 Human Genomics Lab, School of Sport, Exercise and Health Sciences, Loughborough University, Loughborough LE11 3TU, UK

**Keywords:** multifactorial, knee osteoarthritis, genetic models, haplotypes, ROC curve analysis, predictive marker

## Abstract

The present study attempted to investigate whether concerted contributions of significant risk variables, pro-inflammatory markers, and candidate genes translate into a predictive marker for knee osteoarthritis (KOA). The present study comprised 279 confirmed osteoarthritis patients (Kellgren and Lawrence scale ≥2) and 287 controls. Twenty SNPs within five genes (CRP, COL1A1, IL-6, VDR, and eNOS), four pro-inflammatory markers (interleukin-6 (IL-6), interleuin-1 beta (IL-1β), tumor necrosis factor alpha (TNF-α), and high sensitivity C-reactive protein (hsCRP)), along with significant risk variables were investigated. A receiver operating characteristic (ROC) curve was used to observe the predictive ability of the model for distinguishing patients with KOA. Multivariable logistic regression analysis revealed that higher body mass index (BMI), triglycerides (TG), poor sleep, IL-6, IL-1β, and hsCRP were independent predictors for KOA after adjusting for the confounding from other risk variables. Four susceptibility haplotypes for the risk of KOA, AGT, GGGGCT, AGC, and CTAAAT, were observed within CRP, IL-6, VDR, and eNOS genes, which showed their impact in recessive β(SE): 2.11 (0.76), recessive β(SE): 2.75 (0.59), dominant β(SE): 1.89 (0.52), and multiplicative modes β(SE): 1.89 (0.52), respectively. ROC curve analysis revealed the model comprising higher values of BMI, poor sleep, IL-6, and IL-1β was predictive of KOA (AUC: 0.80, 95%CI: 0.74–0.86, *p* < 0.001), and the strength of the predictive ability increased when susceptibility haplotypes AGC and GGGGCT were involved (AUC: 0.90, 95%CI: 0.87–0.95, *p* < 0.001).This study offers a predictive marker for KOA based on the risk scores of some pertinent genes and their genetic variants along with some pro-inflammatory markers and traditional risk variables.

## 1. Introduction

Osteoarthritis (OA) is a debilitating arthropathy due to the continuing degradation of all joint tissue, such as the meniscus, infrapatellar fat pad, and cartilage, along with subchondral sclerosis and synovial inflammation [1]. Several mechanisms participate in the etiopathogenesis of OA, but intra-articular inflammation is the crucial trigger, propagated by pro-inflammatory cytokines in plasma and synovial fluid [2]. Studies have shown that amongst others, interleukin-6 (IL-6), interleukin 1-beta (IL-1β), and tumor necrosis factor alpha (TNF-α) are the prime culprits that play significant roles in the progressive cascade of OA pathology [1,2,3]. After tissue injury, specific cell surface receptors augment the synthesis of these cytokines within synovium, where they collaborate and cause destruction of the cartilage matrix, resulting in hyperalgesia [1,2,3,4]. They also stimulate the synthesis of C-reactive protein (CRP), a significant marker of systemic inflammation; however, its behavior in OA pathophysiology is controversial as most of the reports, if not all, indicate that higher levels of hsCRP are associated with development but not with radiographic progression of OA [5]. The panoply of clinical information indicates that this inflammatory syndicate convenes to exacerbate cartilage degradation, fragmentation, denudation, and subchondral microstructural changes.

Serum and synovial levels of these inflammatory promoters are genetically mediated and transcriptionally regulated [6]. For instance, a single nucleotide polymorphism (SNP) of guanine (G) to cytosine (C) at −174 bp (rs1800795) on the promoter region of the IL-6 gene decreases IL-6 production significantly [7]. Replacement of adenine (G) with (A) (rs2794521) at 717 bp upstream of the CRP gene enhances transcriptional activity, thereby increasing CRP levels. Although the individual effects of these SNPs are important, they behave differently when present with SNPs of other genes, indicating an epistatic relationship in important disease pathways [7]. Vitamin D receptor (VDR) is an immune-regulator and anti-inflammatory molecule, which influences OA pathology through calcium homeostasis, bone turnover, and remodeling [8]. Furthermore, VDR interacts with endothelial nitric oxide synthase (eNOS) to augment transcriptional efficiency of the NOS enzyme, which is upregulated in chondrocytes and is associated with cartilage destruction in OA [9]. Another gene, collagen type 1 alpha 1 chain (COL1A1), is significantly expressed in inflamed synovium, where it promotes synovial fibrosis due to enhanced collagen synthesis in end stage OA patients [10].

In the multifactorial pathology of OA, evidence to delineate a specific marker has amassed, but due to OA’s complexity, such a marker remains elusive. The evolution of OA of the knee (KOA) is slow and takes years to develop; during that period, one must bear a long trail of pain and suffering. A predictive model to identify vulnerable individuals very early based on the molecular factors they possess is required urgently so that the disease process can be managed in a timely manner. Therefore, the present study attempted to examine the role of the interactive effects of twenty SNPs within five inflammatory genes (CRP, IL-6, VDR, eNOS, and COL1A1) jointly with four pro-inflammatory markers (IL-6, IL-1β, TNF-α, and hsCRP) and many risk factors to reveal a predictive model for KOA.

## 2. Subjects and Methods

### 2.1. Subjects

**Figure 1:**
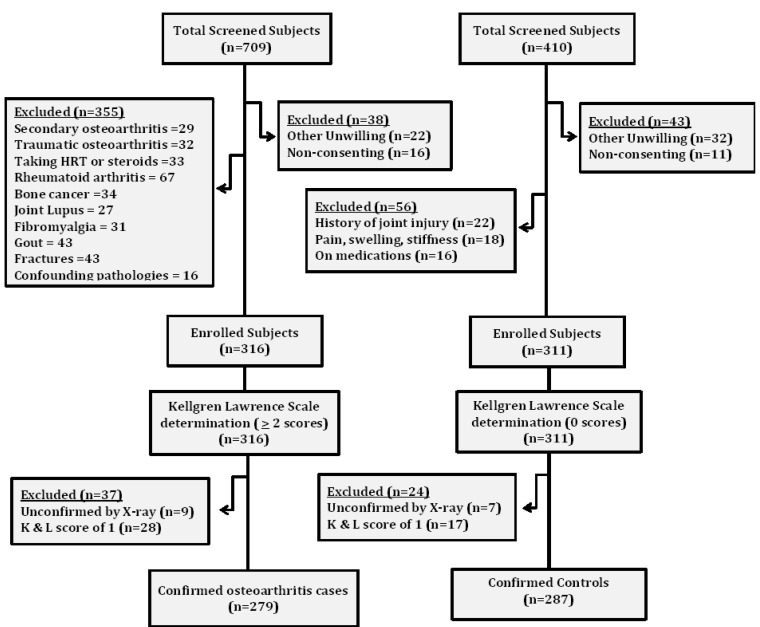
Flow chart showing the data collection protocol.

This study was conducted between August 2016 and August 2020 under the “osteoarthritis predictive marker identification initiative (OPMII)”. After preliminary screening, 279 subjects with moderately severe and severe osteoarthritis verified by radiographic examination of grade ≥2 based on the Kellgren and Lawrence (K&L) scale were enrolled as cases. Matched for age and sex, 287 subjects, who scored zero on the K&L scale and had neither current nor past complaints of skeletal abnormalities or joint pain were selected as controls. Exclusion criteria for the cases included the following: taking hormone replace therapy (HRT) or using corticosteroids; having any type of lupus or suffering from gout; having cancers, rheumatoid arthritis, joint infection/necrosis, fractures, or secondary osteoarthritis; or having any inflammatory disorders or confounding pathologies (osteonecrosis of the medial condyle, psoriatic osteoarthritis, femoral neuropathy, enthesitis, and reactive osteoarthritis). Exclusion criteria for controls included the following: history of joint injury, pain, swelling, or stiffness and/or subjects on medications (Figure 1). Only those subjects who provided prior written consent were enrolled in the study. The privacy of the subjects was preserved to prevent bias. The study protocol was approved by the Institutional Ethical Committee (IEC/350/2017) and stringently followed the Helsinki Declaration.

### 2.2. Sleep Quality and Biochemical Variables

Sleep quality was examined with the Pittsburgh Sleep Quality Index (PSQI), comprising 19 items that identify seven core elements of sleep: sleep quality, how long subjects take to fall asleep, percentage of time in bed, sleep duration, sleep disturbances, medications for sleep, and daytime dysfunction. Global scores of ≥5 indicate poorsleep and <5 good sleep with a sensitivity of 98.7 and specificity of 84.4 for the diagnosis of sleep quality. Total cholesterol (TC), triglycerides (TG), and high-density lipoprotein (HDL) were examined with a one-step enzymatic method (Erba Mannheim, London, UK) with a minimum detection limit of 0.1 mg/L. Low-density lipoprotein (LDL) was evaluated with the Friedewald equation. An Enzyme Linked Immunosorbent Assay (ELISA) was used to determine plasma levels of IL-6, IL-1β, TNF-α, and hsCRP(ThermoFisher Scientific, Waltham, MA, USA), on a microplate reader (Biotek Instruments Inc., Winooski, VT, USA). Diagnostic sensitivities of the kits were <1.0 pg/mL, 0.35 pg/mL, 1.75 pg/mL, and 9.38 pg/mL for IL-6, IL-1β, TNF-α, and hsCRP, respectively. The coefficients of variation for inter- and intra-assay analyses were less than 5% for all these assays.

### 2.3. SNP Selection and Genotyping

SNPs within CRP, IL-6, VDR, eNOS, and COL1A1 genes were carefully selected based on the following criteria: (i) polymorphic with minor allele frequency >5%, (ii) confirmed functional SNP influencing mRNA expression, (iii) significant participant in inflammatory conditions evidenced by published reports, and (iv) validated at NCBI’s refSNP cluster (http://www.ncbi.nlm.nih.gov/snp (accessed on 20 January 2021). Twenty SNPs (Table 1) within these genes were chosen and genotyped. DNA was extracted from the whole blood using the salting-out method. A total of 25 µL of reaction mixture was used to amplify the extracted DNA by employing the polymerase chain reaction (PCR). Amplified DNA was digested with respective high fidelity restriction endonucleases (New England BioLabs Inc., Ipswich, MA, USA). The genotypes were scored and visualized with 2–3% agarose gel electrophoresis depending upon the size of the product. All the experimental work was done without the knowledge of the case/control status to avoid any bias. Fifteen percent of randomly selected samples were repeated for each locus genotyping for replication and validity.

### 2.4. Statistical Power and Population Stratification

Examination of statistical strength of the sample size for inferring genetic associations was checked by using the Power for Genetic Association Analysis (PGA) Package [11]. The minimum detectable relative risk (MDRR) using SNPs and haplotype effects under various models revealed that a sample size of 566 subjects (279 cases and 287 controls) would deliver >85% power to distinguish a minimum genotype relative risk (MGRR) of 2.0 with minor alleles having at least a frequency of 0.15 (rs1800947 in the present sample) at a significance of 0.05. The haplotype relative risk module indicated an MDRR of 1.5 with substantial power (>85%). Population stratification (PS) was tested and the pairwise fixation index (Fst), an indicator of genetic dissimilarity contained in a sub-population, was analyzed by using Arlequin ver. 3.0 software [12]. Fstfor “within population differentiation” was observed to be 0.032 ± 0.017, showing no PS between groups in this study population.

### 2.5. Receiver Operating Characteristic Curve Analysis

To test predictive ability and discriminatory accuracy of biochemical parameters, haplotypes, and traditional risk factors, an area under the receiver operating characteristic curve (AUROC) was modelled with risk scores. Risk scores for biochemical markers and traditional risk factors (TRD) were calculated based on their respective β coefficients (unweighted scores) obtained from logistic regression analysis. Values of these unweighted scores were further standardized by multiplying the lowest absolute value of the coefficient with a number (κ) so that its value becomes 1. All β values were rounded to the closest integer value after multiplying by the value (κ) used for unweighted scores, and their summation represented their respective weighted risk scores. For calculating haplotype-based genetic risk scores, first, individual scores were calculated based on the carriage of risky alleles in the susceptibility haplotype of each gene. Genotypes of risk (R) and non-risk (N) alleles were assigned scores of 0 (NN), 1 (RN), or 2 (RR) for every SNP participating in the respective haplotype. The summation of all SNP-wise risk scores represented the final risk score of every individual. The values deduced as the area under the curve (AUC) from 0.6 to 0.7, 0.7 to 0.8, and 0.8 to 0.9 were considered weak, acceptable, and excellent, respectively.

### 2.6. Statistical Analysis

Differences between proportional and categorical data were analyzed by the chi-square test and continuous data were analyzed with the *t*-test (Student’s *t*) or Mann–Whitney–Wilcoxon rank-sum test. Minor allele frequencies (MAFs) of the SNPs were analyzed by gene counting, and departures from the Hardy–Weinberg equilibrium were evaluated with the Fisher’s exact test. Univariate and multivariable regression analysis were employed to investigate the extent of the independent contribution of variables with KOA. Haplotypes were determined with Arlequin ver. 3.0 software [12]. Two locus epistasis effects (gene–gene) versus risk variables (gene–environment) were analyzed using epiSNP software [13]. Multivariable regression analysis identified a susceptibility haplotype-adjusted model by taking the most prevalent haplotype as the referent. Furthermore, Wald statistics, Akaike information criterion (AIC = 2k−2ln (L)), and uncertainty measure (R^2^*h*) was used to distinguish the best fit model.

## 3. Results

### 3.1. Variables at Baseline and Genetic Correlates

Baseline variables and their differences between cases and controls (Table 1) revealed that data was matched for age (95%CI: 0.74–2.04) and gender (95%CI: 0.72–1.39). No evidence of differences for systolic blood pressure (SBP (95%CI: 0.94–5.85-)), diastolic blood pressure (DBP (95%CI: 0.70–4.38)), total cholesterol (TC (95%CI: 0.92–11.98)), smoking (95%CI: 0.86–1.76), and alcohol drinking (95%CI: 0.79–1.61) were observed between cases and controls. Body mass index (BMI), triglycerides (TG), and low-density lipoprotein (LDL) values were observed to be higher in cases than in controls, and these differences were significantly dissimilar (*p* < 0.001). A higher number of cases were poor sleepers than the number of controls who were poor sleepers (95%CI: 0.09–0.18). Pro-inflammatory markers; IL-6 (*p* < 0.001), IL-1β (*p* < 0.001), and hsCRP (*p* = 0.005) were significantly higher in cases than controls (*p* < 0.001), whereas TNF-α values were observed to be similar between both the groups (*p* = 0.077). MAFs of the SNPs of the CRP gene, i.e., rs2794521 and rs1130864, and the COL1A1 gene SNPs, i.e., rs1107946 and rs1800012, were significantly different between both the groups. MAFs of five SNPs of the IL-6 gene, rs1800795, rs1800796, rs1800797, rs12700386, and rs10499563, were observed to be higher in cases than controls. MAFs for all the three SNPs of the VDR gene, i.e., rs2228570, rs1544410, and rs731236, and four SNPs of the eNOS gene, i.e., rs2070744, rs1799983, rs891512, and rs1808593, differed significantly between both the groups.

### 3.2. Detection of Independent Risk Predictors

Univariate regression analysis of risk variables (Table 2) revealed that BMI (95%CI: 1.64–2.92), LDL (95%CI: 1.37–2.75), TG (95%CI: 1.45–3.15), poor sleep (95%CI: 1.88–3.62), IL-6 (95%CI: 1.73–3.16), IL-1β (95%CI: 1.12–2.83), and hsCRP (95%CI: 1.23–4.16) were significant variables for the risk of osteoarthritis. Binary logistic analysis of these significant risk variables showed that BMI (95%CI: 1.45–2.74), TG (95%CI: 1.31–2.98), poor sleep (95%CI: 1.72–3.09), IL-6 (95%CI: 1.62–3.01), IL-1β (95%CI: 1.07–2.67), and CRP (95%CI: 1.15–3.75) were independent predictors for osteoarthritis risk, whereas, SBP, DBP, TC, and TNF-α did not influence the risk of osteoarthritis.

### 3.3. SNP–SNP Cross Talks, Risky Traits, and Their Modes of Association

The significant single marker effects (*p* < 0.05, *r* > 0.04) were further analyzed to deduce pair-wise epistatic effects with Bonferroni corrections (Table 3). The functional SNP rs1800795 of the IL-6 gene was observed to be highly interactive and showed epistatic relationships with rs1800796, rs1800797, rs12700386, rs10499563, and rs891512, influencing IL-6 (*p* = 0.0003), TG (*p* = 0.0021), BMI (*p* = 0.0013), IL-1β (*p* = 0.0025), and sleep (*p* = 0.0020) through dominant x dominant (DD), interactive (I), dominant x additive (DA), I, and DA effects, respectively. SNP rs1800795 also showed interactions with SNPs rs2228570 and rs731236 of the VDR gene, influencing hsCRP (*p* = 0.0036) and BMI (*p* = 0.0043) through DD and additive × dominant (AD) modes and SNPs rs2794521 and rs1130864 of the CRP gene, influencing sleep (*p* = 0.0017) and IL-1β (*p* = 0.0084) through additive × additive (AA) and AD modes, respectively. Other SNP pairs that showed two-way epistatic relationships were rs1800797-rs18008593, rs1800796-rs1107946, rs12700386-rs1800947, rs2794521-rs1544410, rs891512-rs2070744, and rs2228570-rs1800012, impacting IL-1β (*p* = 0.0035), TG (*p* = 0.0055), hsCRP (*p* = 0.0036), TG (*p* = 0.0014), BMI (*p* = 0.0028), and sleep (*p* = 0.0063) through AD, DA, DD, I, and AA modes, respectively. It is interesting to note that functional SNP rs1800795 of the IL-6 gene interacted with SNPs rs1800796 and rs891512 to influence IL-6 (*p* = 0.037) and sleep (*p* = 0.038) in non-osteoarthritis patients (controls), also through DD and DA modes, respectively. Two locus SNP–SNP epistatic links without risk variables are shown in the figure embedded in the Table 3 to provide a closer look without some of the more complicated interactions.

### 3.4. Haplotype Analysis, Their Risk and Best Fit Mode of Their Manifestation

For haplotype risk analysis of all the genes (Table 4), the most prevalent haplotypes were taken as referents. Haplotypes having frequencies less than five were excluded from further analysis. SNPs of the CRP gene (in the order of rs2794521, rs1800947, and rs1130864) developed into 5 out of 8 possible haplotypes, which captured 85–95% of the genetic variance in osteoarthritis patients and controls. AGT was observed to have a strong association with the risk of osteoarthritis (OR 3.12, 95%CI: 1.88–5.19), which remained significant even after additional adjustment with BMI, TG, sleep, IL-6, IL-1β, and CRP levels (OR 2.73, 95%CI:1.63–4.72). Similarly, SNPs of the COL1A1 gene (in the order of rs1107946 and rs1800012) revealed TG to be a risk haplotype (OR 1.68, 95%CI:1.04–2.70) in the preliminary analysis, but it lost its significance after adjusting for the effects of risk variables. SNPs with the IL-6 gene (in the order of rs1800795, rs1800796, rs1800797, rs2069827, rs12700386, and rs10499563) generated 8 haplotypes out of the expected 64. Haplotype GGGGCT was observed to be risky (OR 2.24, 95%CI: 1.20–4.16) and suggested a susceptibility haplotype (OR 2.10, 95%CI: 1.08–3.79) after adjusting its effect with risk variables. The VDR gene showed 6 haplotypes out of a possible 8 haplotypes, which captured 87–95% of the genetic variance for osteoarthritis patients and controls. The AGC (Bat) haplotype appeared to confer risk (OR 2.10, 95%CI: 1.26–3.93) after adjusting for the effects of risk variables. SNPs within the eNOS gene (in the order of rs2070744, rs1799983, rs1800780, rs391881, rs891512, and rs1808593) developed into 7 out of the 64 possible haplotypes, which captured 78–91% of the genetic variance. Minor alleles of all the SNPs except at position 6 in the form of CTAAAT appeared to be the susceptibility haplotype for osteoarthritis risk (OR 3.12, 95%CI: 1.99–6.72) when its influence was examined after adjusting for the effects of risk variables.

The functional effects of the haplotypes on osteoarthritis risk were further tested more strictly with Wald statistics under dominant, recessive, multiplicative, and general modes of inheritance, and selection of the best fit model was identified with AIC and *R^2^h* (Table 5).It was observed that the susceptibility haplotype AGT of the CRP gene (β ± SE; 2.11 ± 0.76, *p* < 0.001) and the GGGGCT haplotype of the IL-6 gene (β ± SE; 2.75 ± 0.59, *p* < 0.001) influenced osteoarthritis risk in recessive modes, whereas the AGC haplotype of the VDR gene (β ± SE; 2.35 ± 0.65, *p* < 0.001) and the haplotype CTAAAT of the eNOS gene (β ± SE; 1.89 ± 0.52, *p* < 0.001) manifested in dominant and multiplicative modes, respectively.

### 3.5. Predictive Ability of Traditional Risk Factors, Biochemical Parameters, and Haplotypes

**Figure 2:**
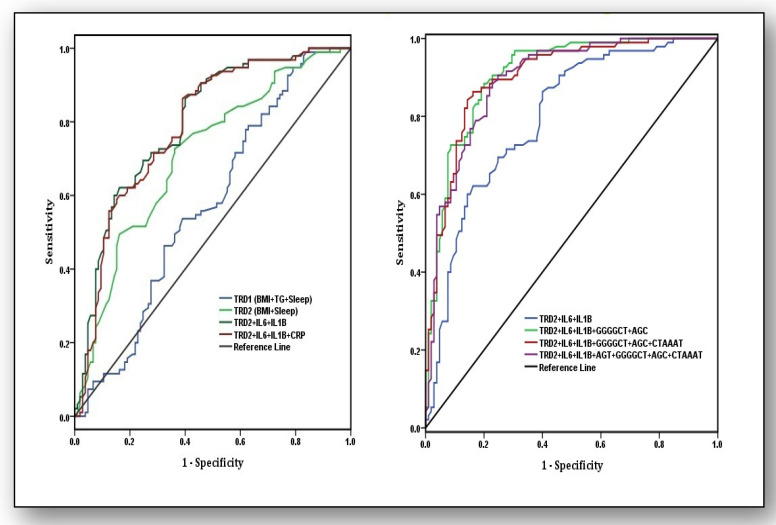
Areas under the receiver operating characteristic (AUROC) curves for the analysis of predictive ability of traditional risk factors (TRD1), pro-inflammatory markers, and susceptibility haplotypes for osteoarthritis risk. Traditional risk factors are body mass Index (BMI), triglycerides (TG), and sleep quality (Sleep). Pro-inflammatory markers are interleukin-6 (IL-6), interleukin 1-beta (IL-1β), and high sensitivity C-reactive protein(hsCRP).Haplotypes are AGT of the CRP gene, GGGGCT of the IL-6 gene, AGC of the VDR gene, and CTAAAT of the eNOS gene. In the first model, values for thearea under curve were as follows: for TRD1: BMI + TG + Sleep (AUC: 0.57 ± 0.040, 95%CI:0.49–0.65), TRD2: BMI + Sleep (AUC: 0.71 ± 0.037, 95%CI: 0.64–0.78), TRD2 + IL-6 + IL-1β (AUC: 0.80 ± 0.031, 95%CI: 0.74–0.86), and TRD2 + IL-6 + IL-1β + hsCRP (AUC: 0.79 ± 0.032, 95%CI: 0.72–0.85). In the second model, TRD2 + IL-6 + IL-1β (AUC: 0.80 ± 0.031, 95%CI: 0.74–0.86), TRD2 + IL-6 + IL-1β + GGGGCT + AGC (AUC: 0.91 ± 0.021, 95%CI: 0.87–0.95), TRD2 + IL-6 + IL-1β + GGGGCT + AGC + CTAAAT (AUC: 0.90 ± 0.022, 95%CI: 0.86–0.94), and TRD2 + IL-6 + IL-1β + AGT + GGGGCT + AGC + CTAAAT (AUC: 0.89 ± 0.022, 95%CI: 0.85–0.94).

Weighted risk scores (effect estimates) were used to measure the predictive power of traditional risk factors (TRD), biochemical parameters, and haplotypes by using AUROC (Figure 2). In the first model, estimation of TRD and biochemical parameters was calculated, and out of these estimates, variables were omitted that did not change the AUC values. Analysis revealed that TRD1 (BMI + sleep + TG) was not predictive (AUC: 0.57, 95%CI: 0.49–0.65) but omitting TG levels in TRD2 (BMI + Sleep) improved the predictive ability significantly (AUC: 0.71, 95%CI: 0.64–0.78). Similarly, predictive strength of the model increased when TRD2 was supplemented with biochemical parameters IL-6, IL-1β, and CRP (AUC: 0.79, 95%CI: 0.72–0.85), which improved further when CRP was omitted in the model comprising TRD2 + IL-6 + IL1β (AUC: 0.80, 95%CI: 0.74–0.86). This model had the most significant predictive strength and was included in a second model with all susceptibility haplotypes, which yielded an AUC of 0.89 (95%CI: 0.85–0.94). When haplotype CTAAAT was omitted, the AUC further improved to 0.90 (95%CI: 0.86–0.94). The predictive strength comprising TRD2 + IL-6 + IL1β + GGGGCT + AGC provedto be the best (AUC: 0.91, 95%CI: 0.87–0.95) for discriminating osteoarthritis patients from controls.

## 4. Discussion

The present study identified a predictive model based on the collective contribution of some traditional risk predictors, relevant biochemical parameters, and genetic candidates for the risk of KOA in the population of Punjab, India. The clinical literature guided our thoughts to consider that in multifactorial pathologies, not a single factor but rather interactions between many “biological culprits” shape the phenotype, otherwise contradictory inferences prevail. For instance, some of the reports suggest that higher CRP levels at baseline are predictive of cartilage loss, synovial inflammation, and progression of KOA [14,15], whereas a meta-analysis showed that neither hsCRP levels nor its gene expression is associated with incidence or progression of KOA without correction for the effect of BMI [16]. Individual SNPs fail to capture genetic variance, whereas haplotypes reflect more robust pictures of these variances and uncover concerted roles of the alleles. None of the studies so far reported CRP-haplotyperelated effects on hsCRP levels in KOA. For the first time, results of the present study highlight that a susceptibility haplotype of AGT of the CRP gene is significantly associated with higher hsCRP levels after adjusting for the effects of BMI, TG levels, and sleep (OR 2.73, 95%CI: 1.63–4.72); however, this result lacked predictive strength so should not be used as a predictive marker for KOA. Inconsistency also exists for the role of the IL-6 gene vis-à-vis KOA, as functional SNP rs1800795 of the IL-6 gene has been observed to correlate strongly with knee OA in a meta-analysis [17], whereas another meta-analysis could not observe it [18]. It is highly likely that transcription control of IL-6 levels by functional SNP rs1800795 is not additive but confers KOA risk by its interaction with other SNPs such as rs1800796, rs1800797, rs2069827, rs12700386, and rs10499563 in a haplotype GGGGCT [7]. The present study affirms that susceptibility haplotype GGGGCT within the IL-6 gene influences higher plasma IL-6 levels but also confers substantial osteoarthritis risk for the knee (OR 2.10, 95%CI:1.08–3.79) after adjusting for the effects of BMI, TG, and sleep.

Vitamin D is a significant mediator of osteoblast proliferation and bone mineralization in addition to its active role in the synthesis of proteoglycans, alkaline phosphatase, and osteocalcin [19]. Therefore, low levels of vitamin D in the body are associated with decreased muscle power strength, poor knee rehabilitation, locomotor dysfunction, and cartilage loss [19]. The T allele of the VDR gene SNP rs731236 has a functional effect on mRNA expression, and lack of this allele poses a three-fold higher risk of KOA [8]. The present study exposed a susceptibility haplotype AGC (Bat) within the VDR gene, and bearers of this haplotype are at a two-fold higher risk of KOA after adjusting for the effect of risk variables (TG, BMI, and sleep). Most of the studies investigating this link did not adjust for the effects with significant confounders, especially sleep quality, which is considerably influenced by vitamin D deficiency. Poor sleep is not an obvious artefact of skeletal abnormalities, as it has strong clinical connotations. For instance, poor sleep induces an immune response of inflammation, which was clarified by a study highlighting that every one hour of sleep deficiency increases 9–12% of the circulating levels of IL-6, TNF-α, and hsCRP levels [20]. VDR interacts epistatically with eNOS to transcriptionally regulate the production of nitric oxide (NO), which is increased in chondrocytes, and its excessive production is autotoxic, which causes tissue and cartilage destruction in OA [9]. Two-way epistatic interactions with 20 SNPs analyzed in the present study highlight that all the functional SNPs of CRP, IL-6, COL1A1, VDR, and eNOS genes participate and interact with other SNPs in influencing risk variables and pro-inflammatory cytokines, which indicates that not an individual SNP but rather SNP–SNP cross talks capture the impact on risky traits.

Some possible limitations of this study may have affected the interpretations. Firstly, gender-specific analysis of knee osteoarthritis has been done owing to the relatively small sample size of men and women in the study. Therefore, additional gender-specific studies with appropriate statistical strength may clarify differences. Secondly, all the explanations are with reference to a population from Punjab; consequently, the findings may not be generalizable to other populations. Hence, such studies in higher cohorts involving other populations and ethnic groups are required to confirm the findings. Finally, despite the current study involving five genes, twenty SNPs, four pro-inflammatory markers, and some pertinent traditional risk factors, further studies including additional microRNAs as epigenetic modulators would be worth pursuing.

In conclusion, the present study observed a predictive model that highlights that traditional risk variables such as BMI and sleep are good predictors for the identification of KOA (AUC: 0.71, 95%CI: 0.64–0.78) and the predictive capacity increased when IL-6 and IL-1β were also involved in the model (AUC: 0.80, 95%CI:0.74–0.86). Likewise, further addition of two susceptibility haplotypes, AGC and GGGGCT, with this model made it an excellent predictor for distinguishing knee osteoarthritis patients from controls (AUC: 0.91, 95%CI: 0.87–0.95).

## Figures and Tables

**Table 1:** Baseline variables of the study participants.

Variables	Cases (*n* = 279)	Controls (*n* = 287)	*p* Value (95%CI)
Age	59.22 (9.41)	58.57 (7.27)	0.357 (0.74–2.04)
Gender (men/women)	138/141	142/145	0.997 (0.72–1.39)
Systolic blood pressure (mmHg)	135.44 (19.52)	133.05 (22.22)	0.175 (0.94–5.85)
Diastolic blood pressure (mmHg)	86.75 (16.32)	84.91 (14.42)	0.155 (0.70–4.38)
Body mass index (Kg/m^2^)	29.40 (4.32)	26.72 (4.45)	<0.001 (1.95–3.40)
Total cholesterol (mg/dL)	168.82 (38.72)	163.29 (39.40)	0.093 (0.92–11.98)
Triglycerides (mg/dL)	169.36 (22.80)	155.72 (28.60)	<0.001 (9.36–17.92)
Low density lipoprotein (mg/dL)	212.30 (35.55)	152.34 (27.39)	<0.001 (54.72–65.19)
Current smokers	92 (32.97)	82 (28.57)	0.256 (0.86–1.76)
Non-smokers	187 (67.03)	205 (71.43)	
Current alcohol drinkers	93 (33.33)	88 (30.66)	0.500 (0.79–1.61)
Non-drinkers	186 (66.67)	199 (69.34)	
Subjects having good sleep	81 (29.03)	219 (76.31)	<0.001 (0.09–0.18)
Subjects having poor sleep	198 (70.97)	68 (23.69)	
**Biochemical parameters**			
IL-6 levels (pg/mL) ǂ	4.7 (1.1, 8.2)	2.3 (0.9, 3.4)	<0.001
IL-1β levels (pg/mL) ǂ	3.8 (1.3, 6.4)	1.5 (0.7, 2.4)	<0.001
TNF-α levels (pg/mL) ǂ	2.1 (1.0, 3.1)	1.4 (0.6, 2.2)	0.077
hsCRP levels (mg/L) ǂ	2.9 (0.9, 4.1)	1.3 (0.7, 1.9)	0.005
**CRP gene/SNPs**			
MAF; rs2794521 †	0.25 ± 0.026	0.14 ± 0.021	0.001
MAF; rs1800947 †	0.15 ± 0.022	0.12 ± 0.022	0.335
MAF; rs1130864 †	0.29 ± 0.027	0.18 ± 0.023	0.002
**COL1AI gene/SNPs**			
MAF; rs1107946 †	0.14 ± 0.021	0.21 ± 0.024	0.029
MAF; rs1800012 †	0.22 ± 0.023	0.16 ± 0.020	0.049
**IL-6 gene/SNPs**			
MAF; rs1800795 †	0.19 ± 0.023	0.36 ± 0.028	<0.001
MAF; rs1800796 †	0.20 ± 0.024	0.28 ± 0.026	0.024
MAF; rs1800797 †	0.19 ± 0.024	0.27 ± 0.026	0.024
MAF; rs2069827 †	0.14 ± 0.021	0.17 ± 0.022	0.325
MAF; rs12700386 †	0.24 ± 0.026	0.17 ± 0.022	0.040
MAF; rs10499563 †	0.16 ± 0.022	0.30 ± 0.020	<0.001
**VDR gene/SNPs**			
MAF; rs2228570 †	0.41 ± 0.029	0.31 ± 0.027	0.012
MAF; rs1544410 †	0.47 ± 0.030	0.35 ± 0.028	0.004
MAF; rs731236 †	0.43 ± 0.030	0.34 ± 0.028	0.029
**eNOS gene/SNPs**			
MAF; rs2070744 †	0.23 ± 0.025	0.16 ± 0.021	0.032
MAF; rs1799983 †	0.22 ± 0.025	0.10 ± 0.018	<0.001
MAF; rs1800780 †	0.45 ± 0.030	0.46 ±0.029	0.811
MAF; rs3918181 †	0.38 ± 0.029	0.33 ± 0.028	0.216
MAF; rs891512 †	0.24 ± 0.026	0.16 ± 0.022	0.019
MAF; rs1808593 †	0.21 ± 0.024	0.14 ± 0.020	0.025

Values are numbers (percentages) or means (SD) except ǂ where values are medians (interquartile range). Values of † are means (SEP; standard error of proportion). *p* values are according to the chi-square test for categorical variables, *t*-test for continuous variables, and Wilcoxon Rank Sum test for pro-inflammatory markers (IL-6, IL-1β, TNF-α, and hsCRP) levels. MAF: minor allele frequency, IL-6:interleukin-6, IL-1β: interleukin 1-beta, TNF-α: tumor necrosis factor alpha, hsCRP: high sensitivity C-reactive protein.

**Table 2:** Univariate and multivariate analysis of various variables associated with osteoarthritis risk.

Variables	Univariate Analysis				Multivariate Analysis			
	Β	Exp (β)	95%CI	*p* Value	Β	Exp (β)	95%CI	*p* Value
SBP (mmHg)	0.718	2.05	0.93–3.15	0.596	----	-----	-----	----
DBP (mmHg)	0.559	1.75	0.87–2.84	0.791	----	-----	-----	----
BMI (kg/m^2^)	0.788	2.20	1.64–2.92	0.002	0.667	1.95	1.45–2.74	0.013
TC (mg/dL)	0.693	2.00	0.90–2.91	0.832	----	-----	-----	----
LDL (mg/dL)	0.615	1.85	1.37–2.75	0.034	0.451	1.57	0.96–2.38	0.058
TG (mg/dL)	1.019	2.74	1.45–3.15	<0.001	0.891	2.44	1.31–2.98	0.007
Sleep (global score)	1.232	3.43	1.88–3.62	<0.001	1.153	3.17	1.72–3.09	0.004
IL-6 (pg/mL)	1.175	3.24	1.73–3.16	<0.001	1.098	3.00	1.62–3.01	0.002
IL-1β (pg/mL)	1.040	2.83	1.12–2.83	0.007	0.920	2.51	1.07–2.67	0.028
TNF-α (pg/mL)	0.625	1.87	0.84–2.85	0.572	-----	-----	-----	-----
hsCRP(mg/L)	0.577	1.78	1.23–4.16	0.003	0.501	1.65	1.15–3.75	0.021

SBP: systolic blood pressure, DBP: diastolic blood pressure, BMI: body mass index, TC: total cholesterol, LDL: low density lipoproteins, TG: triglycerides, IL-6:interleukin-6, IL-1β: interleukin 1-beta, TNF-α: tumor necrosis factor alpha, hsCRP: high sensitivity C-reactive protein. Groups in models are SBP: ≤120 vs. >120, DBP: ≤80 vs. >80, BMI: <25 vs. ≥30 kg/m^2^, TC: ≤200 vs. >200, LDL: ≤100 vs. >100, TG: ≤150 vs. >150>, Sleep quality: global score <5 vs. ≥5, IL-6: ≤3 vs. >3 pg/mL, IL-1β: ≤3 vs. >3 pg/mL, TNF-α: ≤3 vs. >3 pg/mL, CRP: ≤3 vs. >3 mg/L.

**Table 3:** Significant SNP–SNP cross talks and epistasis effects amongst CRP, COL1A1, IL-6, VDR, and eNOS genes.

**SNP**	**SNP**	**Trait**	**Test**	***P*^ost^**	***P*^nost^**	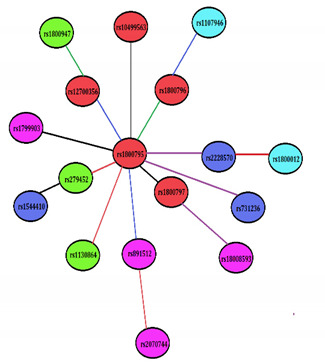
rs1800795	rs1800796	IL-6	DD	0.0003	0.037	
rs1800795	rs1800797	TG	I	0.0021	0.135	
rs1800795	rs2228570	hsCRP	DD	0.0036	1.029	
rs1800795	rs12700386	BMI	DA	0.0013	0.558	
rs1800795	rs10499563	IL-1β	I	0.0025	0.782	
rs1800795	rs2794521	Sleep	AA	0.0017	0.139	
rs1800795	rs1130864	IL-1β	AD	0.0084	0.274	
rs1800795	rs891512	Sleep	DA	0.0020	0.038	
rs1800795	rs731236	BMI	AD	0.0043	0.293	
rs1800797	rs18008593	IL-1β	AD	0.0035	0.536	
rs1800796	rs1107946	TG	DA	0.0055	0.823	
rs12700386	rs1800947	hsCRP	DD	0.0036	0.178	
rs2794521	rs1544410	TG	I	0.0014	0.221	
rs891512	rs2070744	BMI	AA	0.0028	0.135	
rs2228570	rs1800012	Sleep	AA	0.0063	0.835	

*n* the text ng method has been written as suggesteduggested Many SNP–SNP interactions were deduced between osteoarthritis and non-osteoarthritis subjects, but only significant effects are shown here. *P*^ost^: *p* values showing the gene–gene effect (SNP × SNP) influencing risk variables in osteoarthritis patients (*n* = 279), *P*^nost^: *p* values showing the gene–gene effect (SNP × SNP) influencing risk variables in non-osteoarthritis subjects (*n* = 287).Two-locus effects of these SNP pairs indicate AA: additive x additive (Red line color), AD: additive x dominant (Purple line color), DA: dominant x additive (Blue line color), DD: dominant x dominant (Green line color), and I: interactive effect (Black line color). Lines between SNPs indicate pairwise epistasis effect. Colors of the ellipse indicate Red: IL-6 gene, Green: CRP gene, Cyan: COL1A1 gene, Blue: VDR gene, and Magenta: eNOS gene. IL-6: interleukin-6, IL-1β: interleukin 1-beta, hsCRP: high sensitivity C-reactive protein, LDL: low density lipoprotein, TG: triglyceride, BMI: body mass index.

**Table 4:** Haplotypes of CRP, COL1A1, IL-6, VDR, and eNOS genes for the risk of osteoarthritis.

Haplotype	Cases (*n* = 279)	Controls (*n* = 287)	P*^Cor^*	Unadjusted OR (95%CI)	*p* Value	Adjusted OR (95%CI) ^a^	*p* Value
**CRP gene**							
AGC	0.46 (130)	0.54 (155)	0.10	Referent	----	Referent	----
GGT	0.05 (14)	0.07 (21)	0.46	0.79 (0.39–1.63)	0.65	0.77 (0.36–1.53)	0.56
AGT	0.24 (68)	0.09 (26)	<0.001	3.12 (1.88–5.19)	<0.001	2.73 (1.63–4.72)	0.002
GCT	0.09 (26)	0.07 (20)	0.55	1.55 (0.83–2.90)	0.22	1.31 (0.71–2.55)	0.19
GCC	0.06 (18)	0.08 (24)	0.68	0.89 (0.46–1.72)	0.87	0.77 (0.38–1.52)	0.67
**COL1A1 gene**							
GG	0.59 (165)	0.57(171)	0.99	Referent	----	Referent	----
GT	0.20 (55)	0.13 (38)	0.004	1.68 (1.04–2.70)	0.04	1.54 (0.93–2.19)	0.09
TG	0.08 (23)	0.10 (30)	0.65	0.79 (0.44–1.42)	0.53	0.72 (0.38–1.26)	0.47
TT	0.07(19)	0.08 (24)	0.81	0.82 (0.43–1.55)	0.66	0.74 (0.36–1.38)	0.52
**IL-6 gene**							
GGGGGT	0.23 (65)	0.25 (71)	0.95	Referent	--------	Referent	------
CCGCGC	0.08 (22)	0.07 (19)	0.88	1.26 (0.63–2.55)	0.63	1.11 (0.54–2.31)	0.57
CTACAT	0.06 (18)	0.07 (21)	0.95	0.94 (0.46–1.91)	0.99	0.87 (0.43–1.82)	0.82
GGGGCT	0.15 (43)	0.07 (21)	<0.001	2.24 (1.20–4.16)	0.02	2.10 (1.08–3.79)	0.04
CCGTAT	0.09 (25)	0.08 (24)	0.99	1.14 (0.59–2.19)	0.82	1.02 (0.49–2.00)	0.71
CTGCAC	0.07 (19)	0.07 (21)	0.99	0.99 (0.49–2.00)	0.88	0.79 (0.39–1.89)	0.69
CTGTAT	0.08 (22)	0.09 (27)	0.84	0.89 (0.46–1.71)	0.86	0.71 (0.37–1.43)	0.62
CGAGGC	0.07 (21)	0.14 (49)	0.007	0.57 (0.31–1.07)	0.11	0.63 (0.51–1.12)	0.93
**VDR gene**							
GGT (baT)	0.48 (133)	0.51 (147)	0.70	Referent	--------	Referent	------
ATC (BAt)	0.09 (24)	0.09 (27)	0.97	0.98 (0.54–1.79)	0.92	0.83 (0.4301.54)	0.83
GTT (bAT)	0.10 (29)	0.09 (26)	0.90	1.23 (0.69–2.20)	0.57	1.11 (0.57–2.00)	0.47
AGC (Bat)	0.14 (38)	0.06 (17)	<0.001	2.47 (1.33–4.58)	0.005	2.10 (1.26–3.93)	0.01
ATT (BAT)	0.08 (23)	0.07 (19)	0.78	1.34 (0.70–2.57)	0.48	1.13 (0.60–2.28)	0.33
GTC (bAt)	0.07 (19)	0.05 (16)	0.86	1.31 (0.65–2.66)	0.56	1.10 (0.52–2.38)	0.31
**eNOS gene**							
TTAGGG	0.14 (40)	0.19 (55)	0.19	Referent	--------	Referent	------
TTGGGG	0.13 (36)	0.14 (41)	0.93	1.21 (0.66–2.21)	0.65	1.10 (0.55–1.98)	0.52
CGAAGG	0.12 (33)	0.13 (38)	0.91	1.19 (0.64–2.22)	0.69	1.00 (0.47–1.87)	0.48
CTAAAT	0.25 (69)	0.08 (24)	1 × 10^−11^	3.95 (2.13–7.33)	<0.001	3.12 (1.99–6.72)	0.006
CGGAGG	0.10 (28)	0.09 (26)	0.96	1.48 (0.76–2.90)	0.33	1.27 (0.65–2.45)	0.28
CTGGAT	0.09 (24)	0.09 (27)	0.97	1.22 (0.62–2.42)	0.69	1.09 (0.46–2.00)	0.57
TTAGAT	0.08 (22)	0.06 (19)	0.88	1.59 (0.76–3.33)	0.29	1.33 (0.63–2.88)	0.26

Number of subjects having the haplotype are shown in the parenthesis. All haplotypes that had less than 5% frequencies were excluded from the analysis. P*^Cor^*: *p* values were corrected for multiple comparisons (Bonferroni adjustment). Bold faces show the susceptibility haplotype. ^a^ Odds ratios were adjusted with body mass index, triglycerides, poor sleep, interleukin-6, interleukin 1-beta, and high sensitivity C-reactive protein.

**Table 5:** Functional implications of susceptibility haplotypes and their best fit model.

CRP Haplotype AGT					
Model	ǂ β (SE)	Wald Test	*p* Value	*R*^2^h	AIC
Dominant	0.48 (0.53)	0.90	0.510	0.5972	4210.38
**Recessive**	2.11(0.76)	2.77	<0.001	1.0000	2791.39
Multiplicative	−0.31 (0.79)	−0.39	0.389	0.8817	3912.30
General (0 copy)	−0.09 (0.37)	−0.24	0.560	0.9280	4142.49
General (1 copy)	0.70 (0.89)	0.78	0.021	0.9663	3358.40
**IL-6 Haplotype GGGGCT**					
Dominant	0.52(0.64)	0.81	0.629	0.8213	6571.11
**Recessive**	2.75 (0.59)	4.66	<0.001	0.9922	2785.31
Multiplicative	0.20 (0.43)	0.46	0.372	0.7661	4559.90
General (0 copy)	0.19 (0.32	0.59	0.624	0.8317	5216.29
General (1 copy)	−0.07 (0.51)	−0.14	0.721	0.8922	5888.91
**VDR Haplotype AGC**					
**Dominant**	2.35 (0.65)	3.61	<0.001	1.000	4635.58
Recessive	0.28 (0.53)	0.53	0.412	0.780	5128.44
Multiplicative	0.72 (0.39)	1.85	0.782	0.458	5012.48
General (0 copy)	−0.62 (0.41)	−1.51	0.212	0.673	5344.25
General (1 copy)	0.007 (0.24)	0.029	0.991	0.887	4989.16
**eNOS Haplotype CTAAAT**					
Dominant	0.33 (0.67)	0.49	0.562	0.667	7571.20
Recessive	0.58 (0.63)	0.92	0.411	0.540	7255.23
**Multiplicative**	1.89 (0.52)	3.63	<0.001	0.975	6965.78
General (0 copy)	−0.42 (0.33)	−1.27	0.211	0.521	7349.90
General (1 copy)	0.83 (0.34)	2.44	0.689	0.634	7136.29

Models showing values after adjustment for risk covariates: body mass index, triglycerides, poor sleep, interleukin-6, interleukin 1-beta, and high sensitivity C-reactive protein. ǂ Estimated haplotype effect, *p*: asymptotic value, *R*^2^h: haplotype uncertainty measure, AIC:Akaike information criterion. Values in bold face show highest *R*^2^h values and lowest AIC. Recessive (subjects with1 copy are at the same risk as subjects withno copy), Dominant effect (subjects with 1 copy are at same risk as subjects with2 copies), Multiplicative effect (subjects with 1 copy are at an intermediate risk compared to that ofsubjects withno copies or 2 copies).

## Data Availability

Data presented in this paper are available on request from the corresponding author. The data are not publicly available due to ethical issues.
